# Correction: Comprehensive study of resveratrol associated to sulfadiazine in lymphocytes infected with *Toxoplasma gondii*: involvement of purinergic signaling in immune response

**DOI:** 10.1007/s11686-026-01270-9

**Published:** 2026-05-13

**Authors:** Nathieli Bianchin Bottari, Priscila Marquezan Copetti, Bianca Fagan Bissacotti, Taís Vidal, Anielen Dutra Da Silva, Mateus Fracasso, Karine Paula Reichert, Jelson Nauderer, Vera Maria Melchiors Morsch, Cinthia Melazzo, Aleksandro Schafer Da Silva, Maria Rosa Chitolina Schetinger

**Affiliations:** 1 Department of Biochemistry and Molecular Biology, Federal University of Santa Maria (UFSM), Avenida Roraima, nº1000, Camobi District, Santa Maria, RS 97105900 Brazil; 2 Graduate Program in Animal Science, University of Santa Catarina (UDESC), Chapecó, SC Brazil; 3 Department of Microbiology and Parasitology, Institute of Biology, Federal University of Pelotas (UFPel), Pelotas, RS Brazil

**Correction to: Acta Parasitologica (2026) 71:68** 10.1007/s11686-026-01252-x

In the Original publication, article title was wrongly published in Portuguse as:

“Estudo Abrangente do Resveratrol Associado à Sulfadiazina em Linfócitos Infectados com Toxoplasma gondii: Envolvimento da Sinalização Purinérgica na Resposta Imunológica.

It should be in english as:

“Comprehensive study of resveratrol associated to sulfadiazine in lymphocytes infected with Toxoplasma gondii: involvement of purinergic signaling in immune response”

The Original article has been corrected.

